# COFACTOR Drammen dataset - 4 years of hourly energy use data from 45 public buildings in Drammen, Norway

**DOI:** 10.1038/s41597-025-04708-3

**Published:** 2025-03-06

**Authors:** Synne Krekling Lien, Harald Taxt Walnum, Åse Lekang Sørensen

**Affiliations:** 1 Sintef Community, Oslo, Norway; 2 Department of Electric Energy, Norwegian University of Science and Technology, Trondheim, Norway

**Keywords:** Energy supply and demand, Electrical and electronic engineering, Civil engineering, Energy modelling

## Abstract

To limit energy consumption and peak loads with increased electrification of our society, more information is needed about the energy use in buildings. This article presents a data set that contains 4 years (Jan. 2018- Dec. 2021/Mar. 2022) of hourly measurements of energy and weather data from 45 public buildings located in Drammen, Norway. The buildings are schools (16), kindergartens (20), nursing homes (7) and offices (2). For each building, the data set contains contextual data about the buildings including their floor area, construction year, energy label, information about their heating system and ventilation system in addition to time series data of energy use and weather data. For some of the buildings, the energy measurements only contain measurements of hourly imported electricity, while the time series data for other buildings have submeters for different energy services and technologies. Researchers, energy analysts, building owners and policy makers can benefit from the dataset for e.g. hourly load disaggregation, forecasting of energy loads and flexibility, grid planning and modelling activities.

## Background & Summary

Buildings operations account for approximately 30% of global energy sector emissions. Most of these emissions come from direct emissions related to generation of heat and electricity for the buildings^1^. Electrification of buildings’ energy use is recognized as a crucial approach for establishing a more sustainable energy system and curbing emissions contributing to climate change^2^. Norway’s building sector is unique, with its highly electrified heating supply, driven by historically low electricity prices. Consequently, buildings were responsible for around 37% of delivered energy and 55% of delivered electricity in 2021^3^. This significantly impacts peak electricity demand, aligning with the coldest hours of the year. To address anticipated bottlenecks occurring a few hours annually, substantial grid investments are planned for the coming years, with end-users covering the costs through their electricity bills^4^. A central challenge in the years to come is how to curb the growth of peak loads with increased electrification and accurately predict building loads to ensure a secure yet efficient grid. Concurrently, Standards Norway has released a new standard, NS3032, outlining a methodology for calculating building peak loads^5^. To develop accurate methods to predict peak loads of buildings, more information is needed about the electricity use of buildings. This dataset presented in this article is part of a larger data set collection process in the research project “COincidence FACTOR for buildings” (COFACTOR, https://www.sintef.no/prosjekter/2021/cofactor/) which is aiming to provide insights into standard load profiles, coincidence factors, and peak loads for typical building categories in Norway.

Drammen is a municipality located close to Oslo in the south-east of Norway with approximately 104 000 residents. Drammen administrates 32 schools and 36 public kindergartens (https://www.drammen.kommune.no/om-kommunen/organisasjon-administrasjon/fakta-om-drammen/). The data set consists of energy and weather measurements from 45 public buildings - including schools, kindergartens, nursing homes and offices - located in Drammen Norway. The buildings have different heating systems. Some are connected to a district heating grid, while others have electric heating. Several of the kindergartens have only direct electric heating, but no submeters for this energy use. Other bigger buildings in the data set use both electric boilers and heat pumps of which there are separate measurements of electricity use and heat production/heat consumption in the dataset. While several building energy datasets from other countries are available^6–11^, the COFACTOR Drammen dataset provides value for several reasons. It is an open data set with hourly energy measurements from different service buildings, and no other available data sets like this exist for Norway. Spanning over 4 years, the COFACTOR Drammen dataset captures data continuously, capturing the effects of the weather and user behaviour on the energy use. The measurements for each building contain both imported electricity and district heating, outdoor climate and for several buildings – sub meters for different heating appliances and other appliances.

The COFACTOR Drammen dataset has been slightly cleaned of false 0 values and outliers, ensuring uniform and comparable dataset that can be directly used by various researchers with minor alterations. In contrast, data sets that only provide raw data require researchers to individually handle data cleaning, leading to varied approaches, and consequently, this discrepancy hampers the reproducibility and comparison when the data set is later used in research.

The COFACTOR Drammen dataset has been used for several applications, including training of load disaggregation of electric boilers from the total electricity load in schools, training of automatic classification of building category from buildings’ electricity loads. Developing these methods and testing the approaches with real-world datasets is essential in this research domain. Simulated data fails to accurately represent energy use consumption pattens in buildings, as actual energy measurement datasets typically exhibit a level of complexity that is challenging to predict, and research has shown a large discrepancy between energy measurements and simulations in buildings^12^.

**Fig. 1:**
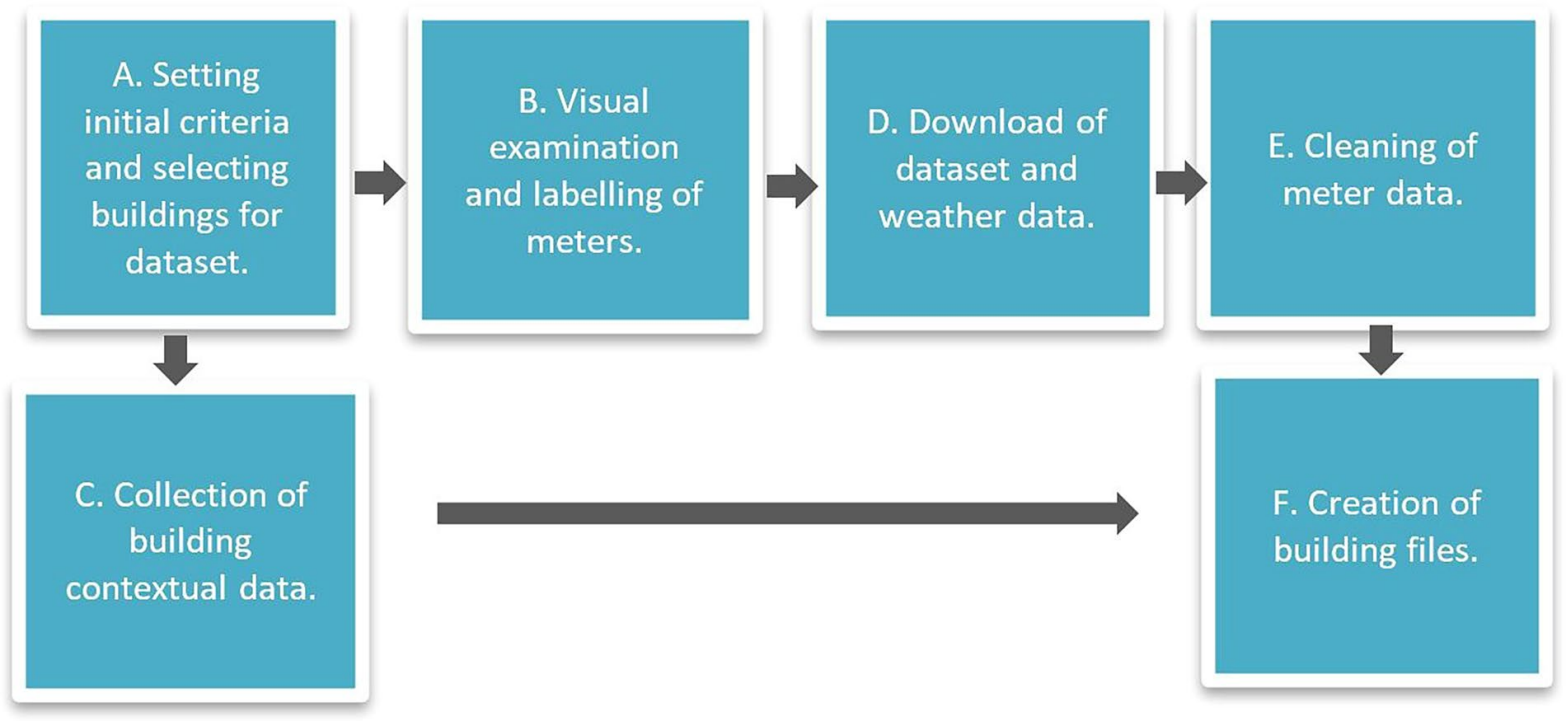
A schematic overview of workflow on data collection in the COFACTOR Drammen Data set.

Figure 1 gives a schematic overview of how data was collected and treated to create the files in the COFACTOR Drammen data set. The following chapters describe how the data set was collected and treated in more detail, how the data is structured and how it can be used for research and analysis.

A. **Setting initial criteria and selecting buildings for dataset:** In cooperation with Drammen Eiendom, a set of buildings in their building portfolio were selected to be included in this data set. Buildings in the categories school, kindergarten, nursing homes and offices were included in the data set, while buildings in less uniform categories, such as sports facilities, parking lots and ambiguous buildings, were not included.
B. **Visual examination and labelling of meters:** All meters were visually inspected to ensure sufficient data quality and that the meters had actual measurements. The meters where then assigned to a meter label according to the purpose/appliance they served.
C. **Collection of contextual building data:** Contextual data about the buildings were collected by going through documentation about the buildings and technical systems as well as through conversations with Drammen Municipality.
D. **Downloading dataset and weather data:** Meters connected to a label were downloaded from the energy surveillance system used by Drammen municipality (Energinet) with hourly resolution. Weather data was acquired for the different building locations. The energy data and weather data were combined to time series data for each building.
E. **Cleaning of energy meter data:** The energy data was cleaned of repeating zero values and outliers.
F. **Creation of building files:** A csv file containing both contextual building data and cleaned time series data was created for every building in the data set. Each file has the same format and time stamp format.

## Methods

### Building characteristics

The data set consists of 45 public buildings located in Drammen Norway. The buildings are classified as schools (16), kindergartens (20), nursing homes (7) and offices (2). The dataset consists of contextual building data and energy and weather time series per building. The time series data have a duration of approximately 4 years (01.01.2018 – 31.12.21/18.03.22) with hourly resolution.

A short description of all buildings available in the dataset is given in Table 1.

**Table 1:** Characteristics of the buildings in the COFACTOR Drammen dataset.

ID	Building category	Year of const-ruction	Heated floor area [m2]	# of users	Space heating source (SH)	Domestic hot water heat source (DHW)	Venti-lation heat source (VH)	Venti-lation Type	Energy perfor-mance Label [A-G]
6396	Kindergarten	1989	437	60	EFH	HWH	EHB	CAV_fxd	Ukn
6397	School	1974	3376	400	GSHP, EB	GSHP, HWH	GSHP, EB	CAV_sch	B
6398	Kindergarten	2007	569	68	ASHP, EB	ASHP, EB	ASHP, EB	CAV_sch	Ukn
6399	Nursing home	2013	2000	16	GSHP, EB	GSHP, HWH	GSHP, EB	CAV_sch	B
6400	School	1977	3958	300	EB, EH	HWH, Ukn	EB, EHB	DCV	F
6402	Kindergarten	1986	188	36	EH	HWH	EHB	CAV_sch	Ukn
6404	School	1903	6570	345	DH	DH, HWH	DH	DCV	G
6405	Kindergarten	1976	501	60	EH	HWH	EHB	CAV_sch	Ukn
6406	Kindergarten	2007	497	73	EFH	HWH	EHB	CAV_sch	Ukn
6407	Kindergarten	1964	771	75	EH, EFH	HWH	EHB	CAV_sch	Ukn
6408	School	1907	5273	400	EB	EB, HWH	EB	DCV	C
6409	Kindergarten	1925	1095	97	GSHP, EB	GSHP, EB	GSHP, EB	CAV_sch,DCV	Ukn
6410	Nursing home	1986	3327	49	EH	HWH	EHB	DCV	C
6411	Office	1914	8424	Ukn	DH, EB	HWH	DH	CAV_fxd	G
6412	Nursing home	1996	4214	39	GSHP, EB	GSHP, EB	GSHP, EB	CAV_sch	D
6413	School	2015	5086	Ukn	GSHP, SC, EB	SC, HWH	GSHP, EB	DCV	Ukn
6414	School	2013	1807	315	GSHP, EH	HWH, Ukn		CAV_sch	D
6415	Kindergarten	1990	258	128	EFH	HWH	EHB	CAV_sch	Ukn
6416	School	2001	8513	600	EB	EB, HWH	ASHP	DCV	C
6417	Nursing home	1991	2609	23	EH	HWH	EHB	DCV	E
6418	School	1974	4616	435	EH, GSHP	HWH, GSHP	EHB, GSHP	CAV_sch	F/D
6419	Kindergarten	1988	363	95	EFH	HWH	EHB	CAV_sch	Ukn
6420	School	1969	6290	350	EH	EB, HWH	EB, EHB	DCV	D
6421	Kindergarten	2013	1429	96	GSHP, EB	GSHP, EB	GSHP, EB	CAV_sch	C
6422	Kindergarten	1984	462	55	EH	HWH	EHB	CAV_sch	Ukn
6423	Nursing home	1988	3250	39	EH, EFH	HWH	EHB	CAV_sch	C
6424	School	1934	5293	450	GSHP, EH	GSHP, HWH	GSHP	DCV	C
6425	Kindergarten	1918	740	50	EFH	HWH	EHB	CAV_sch	Ukn
6426	Kindergarten	1987	206	22	EFH, EH	HWH	EHB	CAV_sch	Ukn
6428	Kindergarten	1987	535	68	EFH	HWH	EHB	CAV_sch	Ukn
6429	Kindergarten	1800	1396	98	DH, ASHP	HWH	EHB	CAV_sch	Ukn
6431	School	1916	3006	177	EB, EH	EB, HWH	EB, EHB	CAV_sch	F
6432	School	1961	5273	170	GSHP, EB	SC, HWH	GSHP, EB	DCV	B
6433	Kindergarten	1997	864	90	EFH, EH	HWH	EHB	CAV_sch	Ukn
6434	Kindergarten	1972	707	75	EFH, EH	HWH	EHB	CAV_sch	Ukn
6436	Nursing home	2009	6774	40	DH	DH, HWH	DH	CAV_sch	E
6437	Kindergarten	1991	576	60	EFH, EH	HWH	EHB	CAV_sch	Ukn
6438	School	1982	6323	500	GSHP, EH	HWH, Ukn	GSHP, EHB	CAV_sch	E
6439	Kindergarten	1974	1144	102	EH	HWH	EHB	CAV_sch	D
6440	School	1935	3062	260	EH, EFH	HWH	EHB	CAV_sch	F
6441	Office	1922	1510	Ukn	EH	HWH	EHB	CAV_sch	E
6442	Nursing home	2009	4997	72	GSHP, EB	GSHP, HWH	GSHP, EHB	CAV_sch	D
6443	Kindergarten	1993	472	45	EH, EFH	HWH	EHB	CAV_sch	Ukn
6444	School	1927	4006	380	EB, EH	EB, HWH	EB, EHB	DCV	D
6445	School	1967	5585	560	GSHP, EB, EH	GSHP, EB	EB, EHB	DCV	C/E

A summary of all contextual data parameters for the buildings, and the explanations/abbreviations for the contextual data is given in Table 2. Abbreviations for heating sources are given in Table 3.

**Table 2:** Description of contextual data in the files.

Contextual data parameter/code	Description	Data type	Example
Header_line	First line of measurements (for reading the csv)	Int	39
location	Post code/city of building	Str	‘Drammen’
year_of_construction	Year of construction	Int	1958
floor_area	Floor area in square meters	Int	5036
number_of_users	Number of users	Int	200
number_of_units	Number of building units	Int	2
number_of_buildings	Number of buildings on the lot	Int	2
building_category	Building category abbreviation. Kindergarten: ‘Kdg’ Nursing home: ‘Nsh’ Office: ‘Off’ School: ‘Sch’	Str	‘Kdg’
energy_label	Energy label	Str	‘A’ to ‘G’
notes	Any notes/additional information about the building and energy data	Str	‘Description’
central_heating_system	Buildings with central heating systems and water borne heat distribution systems.	Int [0,1,2]	0 – No 1 – yes 2 – Unknown
dhw_heat_source	Type(s) of heating technology for hot water heating. See table below for available options.	Str	‘HWH’
sh_heat_source	Type(s) of heating technology for space heating. See table below for available options.	Str	‘A2A,EFH,EH’
ventilation_heat_source	Type(s) of heating technology for ventilation heating. See table below for available options.	Str	‘DH’
snow_melt_heat_source	Type(s) of heating technology for snow melting. ‘ESM’ See table below for available options.	Str	‘ESM’
cooling_source	Type(s) of cooling technology. Central cooling machine: ‘Cen’ Direct expansion: “DX” Free cooling: “Free” District cooling: “DC” Unknown: “Ukn”	Str	‘DX’
ventilation_types	Type of ventilation technology/strategy used. Demand controlled ventilation: ‘DCV’ Calendar schedule: CAV_sch Fixed: “CAV_fxd” Mechanical extract: “Mech” Natural ventilation: “Nat” Unknown: “Ukn”	Str	‘DCV’
ev_chargepoints	The number of charge points and peak power	Str	1 charger with 22 kWp: ‘1:22’
pv	Photovoltaic system. Location, size (kWp) and inverter capacity (kW)	Str	‘Roof:3:3, Wall:5:4’
night_setback	Uses control system with night set back on ventilation/heating	Int [0,1,2]	0 – No 1 – yes 2 – Unknown
lighting_control	Uses control system with timer or other control for lighting	Int [0,1,2]	0 – No 1 – yes 2 – Unknown
timestamp_format	Format for the timestamp in the time series	Str	‘%Y-%m-%dT%H:%M:%S%z’
time_zone	Time zone	Str	Etc/Gmt-1
building_id	Id of the building	Str	‘6431’

**Table 3:** Abbreviations of heating types and which energy services they are available for.

Option name	Option description	Available for energy services
EB	Electric boiler	SH, DHW, VH, SM
EFH	Electric floor heater	SH
EH	Electric heater	SH
DH	District heating	SH, DHW, VH, SM
GSHP	Ground source heat pump	SH, DHW, VH, SM
ASHP	Air source heat pump	SH, DHW, VH, SM
SC	Solar collector	SH, DHW, VH, SM
HWH	Hot water heater	DHW
EHB	Electric heating battery	VH
Ukn	Unknown	SH, DHW, VH

SH = Space heating, DHW = domestic hot water heating, VH = ventilation heating, SM = Snow melt systems.

All building files include meters weather parameters (outdoor temperature, global radiation, wind direction and wind speed). In addition, they contain different measurements of energy use. The available energy meters for each building are listed in Supplementary Table 1. Abbreviations found in these columns are given in Table 4.

**Table 4:** Abbreviations and explanations about time series column headers/meter types.

Column name	Description	Measurement category	Unit
TimeStamp	Time stamp in local UTC time	Time	Time
Tout	Temperature Outdoor	Weather variable	C
SolGlob	Global Solar Horizontal Radiation	Weather variable	W/m2
WindSpd	Wind speed	Weather variable	m/s
WindDir	Wind direction	Weather variable	deg
ElPV	Electricity production	Electricity production from PV	Wh
ElImp	Electricity imported	Electricity Import	Wh
ElLight	Electricity lighting	Electricity use	Wh
ElLightOut	Electricity outdoor lighting	Electricity use	Wh
ElFan	Electricity ventilation fans	Electricity use	Wh
ElOth	Electricity other	Electricity use	Wh
ElDHW	Electricity for domestic hot water heating	Electricity use	Wh
ElBoil	Electricity for electric boiler	Electricity use	Wh
ElHP	Heat pump	Electricity use	Wh
ElTech	Electricity for technical room/server room	Electricity use	Wh
ElSnow	Electricity for snow melt	Electricity use	Wh
ElHWH	Electricity for hot water heater	Electricity use	Wh
ElClComf	Electricity for comfort cooling	Electricity use	Wh
ElClTech	Electricity for technical cooling	Electricity use	Wh
ElPump	Electricity use for pumps	Electricity use	Wh
ElVentPool	Electricity for ventilation of pool area	Electricity use	Wh
ElEV	Electricity for charging of electric vehicles	Electricity use	Wh
HtOil	Heat production from Oil	Heat production	Wh
HtSC	Heat production from Solar collector	Heat production	Wh
HtDH	Heat from district heating	Heat Import	Wh
HtTot	Heat Total HtTot = HtSpace + HtDHW	Heat use	Wh
HtSpace	Space heating HtSpace = HtRoom + HtVent	Heat use	Wh
HtRoom	Room heating	Heat use	Wh
HtVent	Ventilation heating battery	Heat use	Wh
HtDHW	Domestic Hot Water	Heat use	Wh
HtHP	Heat from heat pumps	Heat production	Wh
HtSnow	Snow melting	Heat use	Wh
HtPool	Heat for pool heating	Heat use	Wh

### Contextual building data

All building files contain contextual data about the building, which describes their type, users, and technical systems. The availability of contextual data varies between the buildings in the dataset. For some buildings, very detailed information is available, while there is less information available for others. For buildings with more sub-meters, more details are usually available. The contextual data consists of several predetermined parameters (codes) and a value for each parameter. Table 2 provides a summary of all available contextual data present in the building files, including their codes/parameters, a description of the contextual data parameters, the data type, and the format/available answers for each parameter

The contextual data about the buildings were collected through conversations with the building owners, by examining internal reports about the buildings and their energy systems and contextual data stored in Energinet. In addition, contextual data for the buildings was found by examining their Energy labels. Energy labels for public buildings are mandated by law^13^ available through the energy label portal (https://portal.ems.enova.no/direct-login) by searching for the building addresses and requires Norwegian BankID for authentication.

### Heating system options

Buildings use different technologies for heating/heating purposes. Each building’s contextual data includes details regarding the heating technologies utilized for four distinct energy services: heating of domestic hot water (DHW), space heating (SH), ventilation heating (VH), and snow melting (SM). The buildings in the data set all use one or multiple heating technologies for these heating energy services. The available options for heating technologies for these services are outlined in Table 3.

### Time series data

To be able to compare measurements of energy purposes from different buildings, building meters have been assigned to predefined measurement purposes. All meter types in the time series data present in the building files are described in Table 4.

### Collection of raw data

The energy use of all public buildings in Drammen is monitored in Energinet which is an administration software tool for energy, waste and environmental surveillance (https://kiona.com/no/produkter/energinet). The types and number of energy meters varies a lot from building to building. The raw data set was downloaded from Energinet in February/March of 2022. At that time, 123 buildings were monitored by Drammen Eiendom in Energinet. 40 of these buildings had only recently been added to Energinet due to a municipality merger and these buildings were left out of this dataset. Only buildings within the categories schools, kindergartens, nursing homes, and offices were considered relevant for the project COFACTOR and are included in the data set. Other buildings, such as sports facilities, energy centrals, fire stations and parking facilities were not included. In total, 24 buildings were discarded due to their building category. 5 buildings were not included as their energy use data was used in a contest (https://adrenalin.energy/Load-Disaggregation-Challenge-Energy-use-in-buildings), and will be published elsewhere. 9 buildings within the desired categories were also discarded due to data quality reasons, due to missing/faulty data, or inability to retrieve required contextual building data. In total, raw data was collected for 45 buildings from Drammen.

### Labelling of meters in the final dataset

The availability of measurements within a building depends on its metering structure. Some buildings adhere closely to the detailed metering structure outlined in SN-NSPEK 3031:2023^14^ or energy purposes, while others may have alternative structures with varying levels of coherence in naming conventions. To compare energy measurements across different buildings, these measurements need to be categorized into predefined purposes. There are various methods to aggregate measurements for different purposes, but this process inevitably leads to some loss of information. Given that all potential use cases for the data may not be initially apparent during collection, it is preferable to maintain as much disaggregation as possible while adhering to standardized purposes. The process of labelling each meter for every building in Energinet was a manual process which required to go into every single meter and check the quality of the data. In many cases there would be meters available for different purposes but there would be data missing for large periods of time. In these cases the meters were not included in the final datasets. Some meters were disregarded due to uncertainty of double counting in the installed meters. For all buildings in the final dataset, weather data and total imported electricity (ElImp) are included, while the number and types of submeters varies between the buildings.

### Cleaning of the energy time series

The cleaning process for the dataset involved several steps to ensure data consistency and accuracy:

- The raw data from each meter was checked against the standard unit of measurement specified in the table. If the unit in the raw data differed from the standard unit, it was converted to the desired unit to maintain consistency across all measurements.
- If any meter had more than a predefined number, denoted as “X”, of missing values within a day, the entire day of measurements was removed. This step helps in maintaining data integrity and ensures that incomplete or unreliable data does not skew the analysis.
- Values that exceeded a certain outlier threshold were identified and removed from the dataset. The threshold for outliers was manually set for each meter based on the specific characteristics of the data. Any values surpassing this threshold were replaced with empty cells to indicate missing or invalid data points.
- Any negative values were replaced with empty cells to eliminate inconsistencies and errors resulting from incorrect readings or recording anomalies.

By following these steps, the dataset was cleaned to remove inconsistencies, missing values, outliers, and erroneous data, thereby preparing it for further analysis and interpretation.

### Weather data

Metrological weather data has been collected for the geographical locations of the buildings from the MET Nordic dataset (Rerun archive version 3) from Norwegian Meteorological Institute (https://github.com/metno/NWPdocs/wiki/MET-Nordic-dataset). The Location of the buildings was defined by their zip-code and the geographical coordinates are collected from a database created by Erik Bolstad (https://www.erikbolstad.no/postnummer-koordinatar/?postnummer=0010). The post code of the buildings were used when downloading the weather data for the exact location, but has not been included in the dataset due to anonymization of the buildings.

Table 5 shows the collected weather data, with the name in the dataset and the corresponding name in the MET Nordic dataset.

**Table 5:** Weather data parameters and corresponding name in source database.

Name	Explanation	Unit	MET Nordic name
Tout	Temperature Outdoor	C	air_temperature_2m
SolGlob	Global Solar Horizontal Radiation	W/m2	integral_of_surface_downwelling_shortwave_flux_in_air_wrt_time. Shifted backwards by one hour to align with “left label”.
WindSpd	Wind speed	m/s	wind_speed_10m
WindDir	Wind direction	deg	wind_direction_10m

## Data Records

The COFACTOR Drammen dataset is available for download from the COFACTOR Community on Zenodo.org: 10.5281/zenodo.11060088^15^ with this section being the primary source of information on the availability and content of the data being described.

**Fig. 2:**
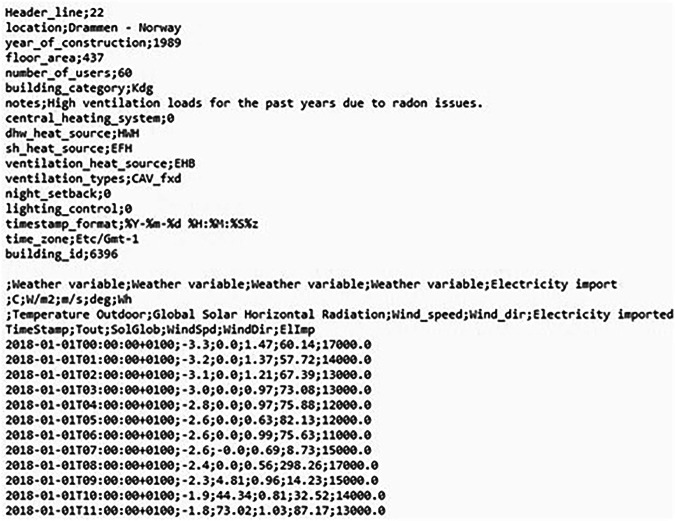
Screenshot of the file for one of the buildings. The file contains contextual data about the building and time series data for the building’s energy use and weather at the building site during the same time period.

The dataset consists of one semicolon-separated csv file per building listed in Table 1– in total 45 csv files. The csv files are all labelled “building_xxxx” as given in the table. The files are structured as shown in Fig. 2, and contain both contextual data and timeseries data. The first line of the csv states the Header_line of the time series. In this example, the time series header is at line 22 and the time series values begin at line 23. An explanation of the time series (with units, short names and full names) is found right above the header line. The contextual data are written above the time series data and header line.

The TimeStamp is in the time zone Etc/Gmt-1 and given on the format “%Y-%m-%dT%H:%M:%S%z”. which is left-oriented (Eg. The time 2018-01-01T00:00:00 means the hour 00:00-01:00). The actual local time zone of the location is “Europe/Oslo”.

The time series have one row per hour of measurements. The time series data contain both the weather data (Tout, SolGlob, WindSpd, and WindDir) and energy meters.

## Technical Validation

### Quality of the data series

The quality of the energy time series is e.g. related to the accuracy of the energy meters. Table 6 evaluates the reliability of the meters for electricity and heating.

**Table 6:** Evaluation of the reliability of the energy meters.

Energy carrier	Level	Quality evaluation
Electricity	Main meter	The main meters for electricity are AMS meters (Automatic Metering System), which are digital electricity meters connected to the electricity provider and used for billing purposes. The quality of AMS-meters is regulated by Norwegian law in^17^. These meters offer remote, real-time monitoring, usually with hourly measurements. They are assumed to have high accuracy and high metering resolution. A building may have more than one AMS-meter installed. In these cases, the measurements from all AMS meters are added together in the column “ElImp”. Additionally, in some cases, electrical boilers may have their own AMS-meters. In these instances, these meters are added as both a sub-meter (“ElBoil”) and summed together with other AMS meters present in the building under “ElImp”.
	Sub-meters	Electricity sub-meters include both AMS-meters for specific appliances (such as electric boilers) and other sub-meters installed in the building. The data quality and labelling of different sub-meters in a building can be varying. Currently, there are strict requirements in place for collection, validation and submission of data from the customers’ main meters (AMS-meters), but there are no such data quality requirements for submeter data. All sub-meters included in the dataset have been manually controlled before added to the data set. Sub-meters with large portions of missing data, or “suspicious” measurements with strange patterns, were not included in the final data set. There are still missing data points in many of the sub meters in the data set.
District heating	Main-meter	The quality of main meters for district heating is regulated by Norwegian law in in^18^. The pretext was updated January 1st, 2023 with quality requirements during use. The measurements from district heating main meters in the dataset are collected before these requirements were put into action. The district heating main meters are used for billing, and the reliability is better for the main meters than for the sub-meters. Comparing AMS and district heating reliability, the reliability for AMS meters is better than district heating meters, due to technical challenges when metering heat flow and temperatures.
Heating	Sub-meters	The quality of sub meters for heating (both from district heating and other heating appliances) is assumed to be very varied. The worst-quality sub meters have been excluded from the data set, but users should still be aware of this varied reliability when working with heating sub-meters in the data set. Having zero-sum quality checks for heating sub meters are not required for Norwegian buildings, and hence, submeters for heating may be measuring overlapping energy purposes. Additionally, there are no requirements or regulations present for sub meters for heating (as there are with AMS-meters and district heating meters).

Data from main meters (AMS-meters) and district heating meters (where available) are generally considered trustworthy due to their high precision and reliability. These meters are typically installed to meet strict industry regulations, ensuring accurate measurements of energy consumption or production for billing of costumers.

On the other hand, data from sub-meters, while still valuable, may require additional evaluation before being used in further research or analysis. The reliability of sub-meter measurements can vary. Therefore, it is essential to assess the quality of data from sub-meters on a case-by-case basis. In some instances, certain periods of measurements from sub-meters may need to be discarded if they are deemed unreliable or if there are indications of measurement errors. The same considerations should be made when downsampling the data, as periods of missing data could affect the outcome.

**Fig. 3:**
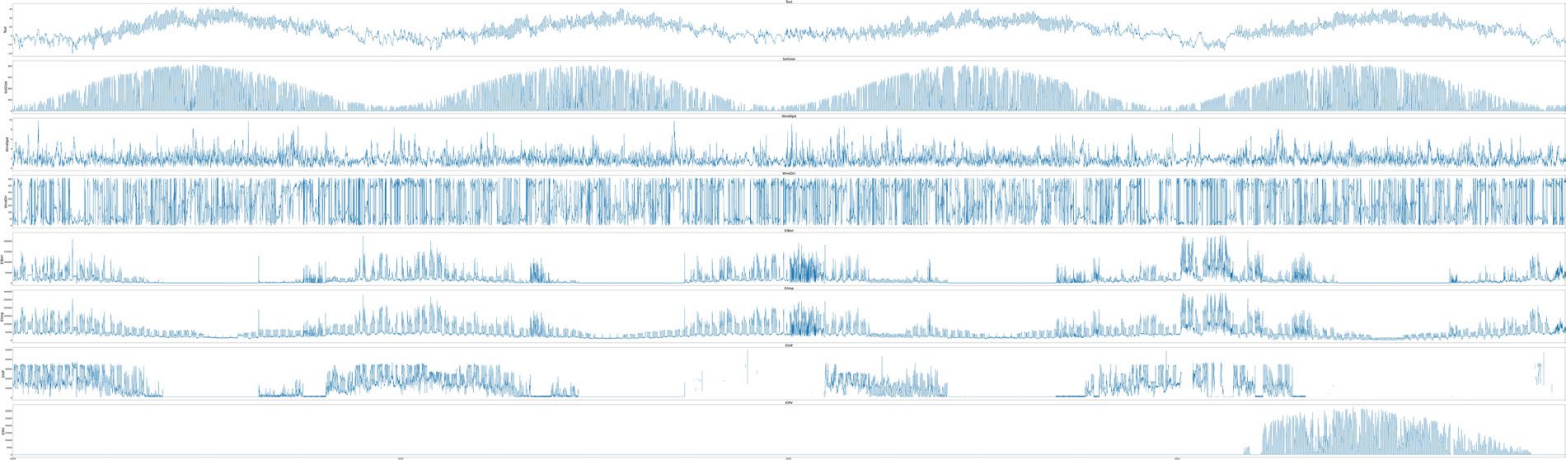
building_6397, a school building with ground source heat pump, electric boiler and PV-panels.

An example of how the quality of main meters and sub meters may vary is shown in Fig. 3 which illustrates all meter values for building_6397, a school equipped with a ground-source heat pump, electric boiler, and PV panels. The figure depicts hourly measurements of weather data alongside readings from energy meters. The electricity meters ElImp (total import) and ElBoil (electricity for the electric boiler, measured by an AMS-meter), exhibits complete data series with minimal errors and periods of missing data. The heat pump meter (ElHP), with measurements collected from a sub-meter and not an AMS-meters, displays periods of missing data. While some periods of missing data are expected, such as during summer months when the school is closed and thermal demand is negligible, sudden missing values during other periods likely stem from measurement errors. Regarding the electricity generation meter (ElPV), missing values are observed until the final year of the measurement period. This is due to the PV panels not being installed until 2021.

As we can see here, the submeters still contain valuable information, but not for the whole measurement period. If this data is to be used in for example training of disaggregation algorithms or other machine learning problems, it may be beneficial to extract only the periods from the dataset where measurements are available for all meters.

Supplementary Table 1 provides a detailed overview of the energy meters included in each building file. It includes the proportion of the time series during which each meter contains data and the sum of values for all columns representing energy consumption. Additionally, it presents results for an artificial meter, “ElRest”, which calculates the hourly difference between the imported electricity (“ElImp”) and the total electricity consumption recorded by the sub-meters. If the photovoltaic electricity generation (“ElPV”) is present, it is added to the imported electricity in the calculation. The purpose of “ElRest” is to show the share of total electricity consumption that is not captured by the sub-meters. However, for some buildings, there are instances where “ElRest” contains negative values. These anomalies may result from missing data in the main meter, incorrect readings in the sub-meters, or overlapping measurements across sub-meters in buildings with several sub-meters. Users are advised to use caution when analysing the buildings and meter readings where this occurs.

### Collection of contextual building data

The contextual data of each building connected in Energinet was collected and reported by Drammen Municipality in 2018 in an internal report. The report was created for internal use in Drammen Municipality and describes the size, location and use of every building, as well as their heating and ventilation systems and control system. The information of this report was used and extracted to fill into a standardized format of contextual data for each building file. In addition, information about the buildings were extracted from the energy labels of the buildings. The energy labelling of buildings is mandated by law for all public buildings larger than 250 m^2^ of heated floor area^13^. The law sets requirements for the competence of professionals who label buildings, but as an energy label is valid for 10 years, there may have been changes in the building information not mentioned in the energy labels. As a control, during the data collection in 2022, the building contextual information from the various sources was reviewed and updated through discussions with Drammen Municipality.

### Practical applications of the dataset

The dataset, either used as a whole or parts of it, have potential for several applications, including:

#### Load profile analysis

The dataset includes electricity main meter readings (AMS) for nearly four full years across all buildings, making it possible to create and analyse load profiles for the buildings and building categories. These profiles show how different building categories, heating technologies, and construction years affect annual and daily energy consumption patterns, and how they are affected by the outdoor temperature and seasonality. By examining these load profiles, we can better understand how energy use varies by building type and season. Load profiles for different building categories can be useful for grid and area planning and provide insights into peak load times, which can aid in better forecasting and planning for energy needs.

#### Sub-meter analysis

Some of the building files include sub-metered data for various energy purposes and appliances. This data provides valuable insights into how different energy uses contribute to the peak loads of buildings and helps assess the demand response flexibility of individual loads.

#### Load disaggregation training

Load disaggregation, also known as non-intrusive load monitoring (NILM), is the process of breaking down a building’s total electricity consumption into its component loads, such as individual appliances or energy purposes. It is a cost-effective alternative to installing sub-meters in buildings, where the electricity for different components are estimated from a single point of measurement, eg. the electricity main meter. Load disaggregation typically relies on data-driven methods which require training data from buildings with sub-meters. This dataset contain energy use measurements for several sub-meters, including 14 buildings with separate meters for electric boiler (ElBoil), but also several other sub-meters from different buildings.

#### Classification training

Classification training involves using energy use data alongside contextual building information to train models that estimate specific characteristics of a building. For instance, this dataset has been used, together with additional building and weather data, to train models capable of predicting building category and heating type based solely on energy time series data^16^. The dataset can be used for training classification models to infer other contextual building attributes as well.

## Usage Notes

The 45 buildings are located within the old geographical Drammen municipality, which is an urban area. The building managers in Drammen Municipality have over a long period been working on energy use monitoring, quality assurance and documentation of their buildings’ energy systems. The selected buildings represent a range of building types, with variations in size, construction year, and heating technologies, offering diverse energy time series data within the different building categories. However, while the dataset reflects variations between different non-residential buildings in Norway, caution should be taken when generalizing the findings to the broader Norwegian building stock, as these buildings are all from the same geographical area, are operated by the same building managers, and have been monitored more closely than other buildings in other parts of the country. The buildings in the dataset may not capture the full range of variations of buildings within the chosen building categories in Norway. The exclusion of certain buildings due to data quality issues could impact the generalizability of the dataset, as those excluded buildings may have different characteristics that are not represented in the final dataset. Despite these limitations, the dataset provides useful insights into energy use patterns for schools, nursing homes, offices and kindergartens in southern-Norway.

Users of this dataset should exercise caution when down sampling, particularly for sub-meter data due to missing data points. Analysing energy use on a daily, monthly, or yearly basis for meters with a significant number of missing data points may lead to a false impression that energy consumption is lower than what it actually is. It is important to assess the data quality of each meter before resampling by referring to Supplementary Table 1 and conducting a visual inspection of the data.

Additionally, users should be mindful of potential bias in the variability of the data. While obvious false zeros and outliers have been removed, there are no control meters to verify errors in the individual meters. As a result, not all remaining data points can be fully validated.

All buildings have main meter data (ElImp) which is considered to be of high quality for the majority of the time series duration, with few missing data points. These meters can be used for a variety of purposes. When using sub-meter data, the user should be cautious about which buildings, meters and time periods are used, depending on the purpose of the analysis.

## Supplementary information

Supplementary Table 1

## Data Availability

No custom code has been generated or is available for this data.
